# Supplementary material for: Imaging quasiperiodic electronic states in a synthetic Penrose tiling
Source: Nat Commun. 2017 Jun 22;8:15961. doi: 10.1038/ncomms15961 (PMC5489715; doi:10.1038/ncomms15961)
Supplement: Supplementary Information [file ncomms15961-s1.pdf]

File name: Supplementary Information
Description: Supplementary Figures, Supplementary Notes and Supplementary References

File name: Peer Review File
Description:

Supplementary Note 1

Additional Conductance Maps and Fourier Transforms

In Fig. 2 of the main text, we demonstrated that the electrons in our synthetic Penrose tiling quasicrystal form a standing wave resonance at the B-sites at the Cu surface states Fermi energy (bias voltage $V = 0$ mV). We have also shown that the brightest peak in the Fourier transform of the conductance map at this energy (map shown in Fig. 2b and again in Supplementary Fig. 1c; Fourier transform shown in Fig. 2e and again in Supplementary Fig. 1h) corresponds to the repetition of the B-sites. To further illustrate the connection between the bright peaks in the Fourier transform of the conductance map and the standing wave resonances at different energies and sites of our synthetic Penrose tiling quasicrystal, we include normalized differential conductance maps at several more energies along with their respective Fourier transforms in Supplementary Fig. 1. It is clear from these conductance maps that the electrons in the Cu surface states form standing wave resonances at different sites at different energies, and accordingly the bright peaks in the Fourier transforms of these maps highlight different resonances as well.

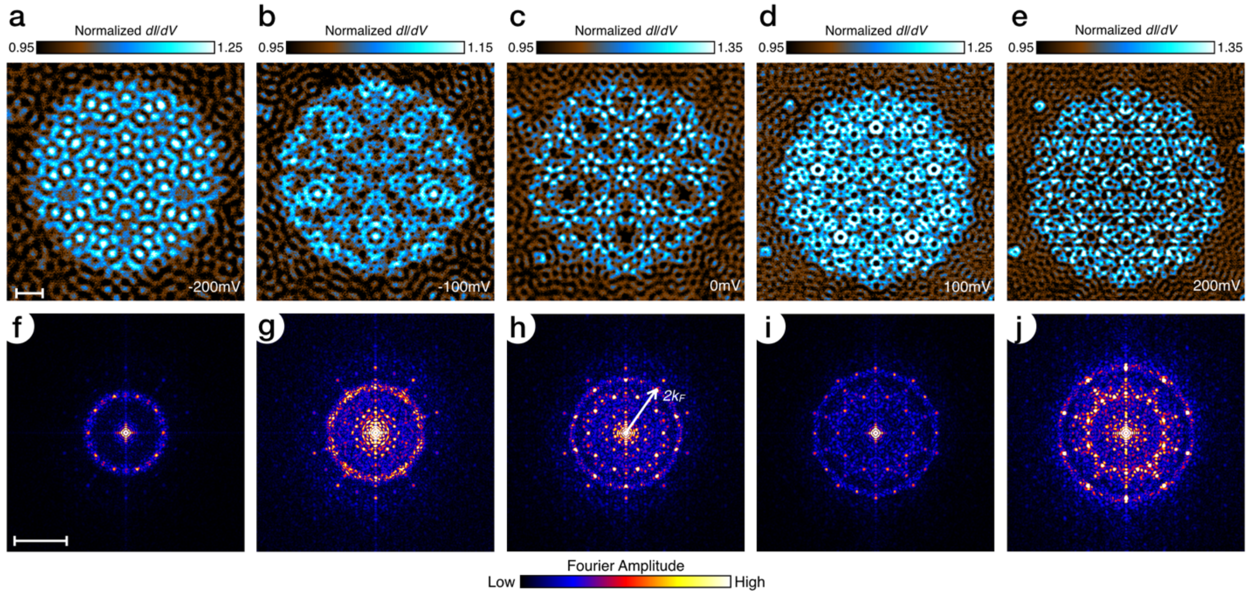

Supplementary Figure 1: Visualizing More Quasicrystalline Electronic States (a - e) Normalized differential conductance maps over a 42 nm by 42 nm field of view measured at bias voltages V = -200 mV, -100 mV, 0 mV, 100 mV, and 200 mV respectively. Scale bar, 5nm. (f) Fourier transform of the conductance map displayed in (a). Scale bar, 4 nm^{-1}. (g) Fourier transform of the conductance map displayed in (b). (h) Fourier transform of the conductance map displayed in (c) with the arrow representing a wave-number value equal to twice the Fermi wave-number of the bare copper surface states (k_F). (i) Fourier transform of the conductance map displayed in (d). (j) Fourier transform of the conductance map displayed in (e).

Supplementary Note 2

Extended Analysis of Electronic States Localized at the B-sites

In Fig. 2f of the main text, we showed a Fourier transform of a model Penrose tiling with tens of thousands of sites (shown again in Supplementary Fig. 2a) to compare to the Fourier transform of our conductance map shown in Fig. 2e. To further explore the origin of the brightest peaks in the Fourier transform, we also took a Fourier transform of a model Penrose tiling with only the B-sites, since those are the sites that are the brightest in the conductance map at the Fermi energy (0 mV) and should therefore correspond to the brightest peaks in the Fourier transform. As you can see in Supplementary Fig. 2b, the brightest peaks in the Fourier transform of the model Penrose tiling with just the B-sites matches the brightest peaks in the Fourier transform of the model Penrose tiling with all of the sites. We also show the Fourier transform of a model Penrose tiling with just A-sites in Supplementary Fig. 2c, where we don't see bright peaks in the same positions as the other Fourier transforms, to demonstrate that this effect is not simply an artifact from excluding several of the sites in the Penrose tiling but comes from the quasi-periodic arrangement of the B-sites in the Penrose tiling. Therefore, this resonant state, evidenced by the bright peaks in the Fourier transform, corresponds to the quasi-periodicity of the B-sites.

In the main text, we claim that the different first order vertex structures can be distinguished electronically within our system. To prove this claim, we examine the electronic behavior of the B-sites further by analyzing them according to their second order vertex structure using the definitions shown in

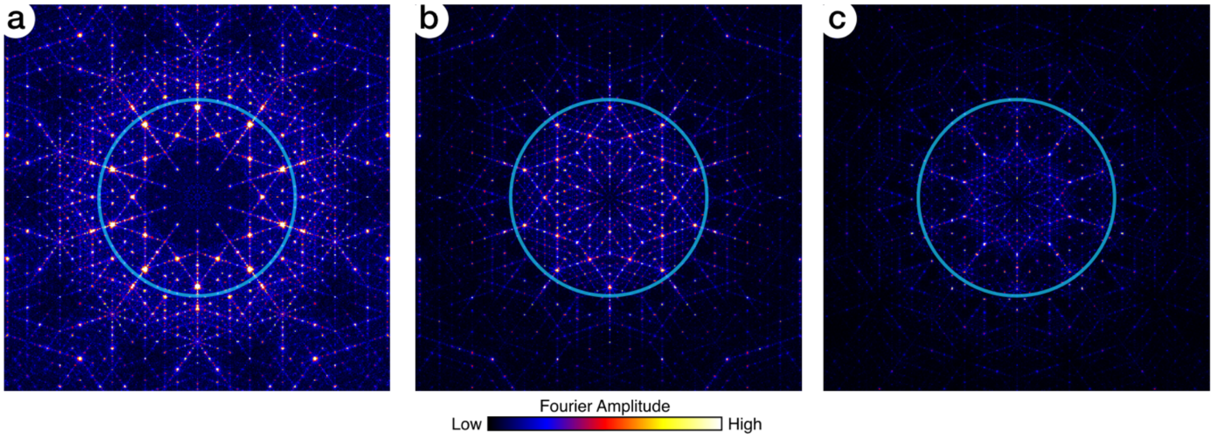

Supplementary Figure 2: Fourier Transforms of Model Penrose Tilings (a) Fourier transform of quasicrystal model structure built with the same proportions as the one used in the experiment but with tens of thousands of sites to improve sharpness of Fourier peaks. The overlaid cyan circle lies just outside a radius of $2k_F$ to highlight the brightest peaks, and is the same circle overlaid in (b) and (c).
(b) Fourier transform of a model Penrose tiling with only the B-sites, again using tens of thousands of sites to improve sharpness, with a cyan circle overlaid just outside a radius of $2k_F$ to highlight the brightest peaks. (c) Fourier transform of a model Penrose tiling with only A-sites, using tens of thousands of sites with a cyan circle overlaid just outside a radius of $2k_F$ to compare to the other Fourier transforms.

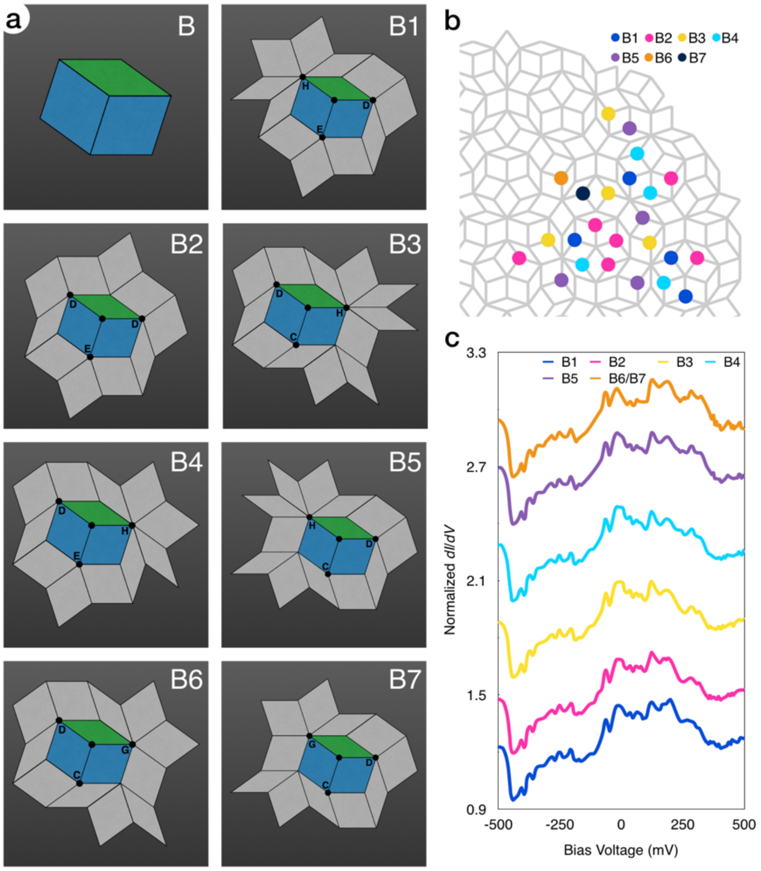

Supplementary Figure 3: Second Order Vertex Structure for the B-sites (a) The 7 possible second order vertex structures for B-sites with nearest neighbor site types labeled. (b) The gray diagram illustrates the Penrose tiling assembled in our experiment. The color dots mark the classification of each B-site where we measured the differential conductance spectra. (c) Normalized conductance spectra averaged by second order vertex type (where B6 and B7 have been averaged together since they are mirror images of each other, and there is only one spectrum for each type). The y-scale refers to the bottom spectra taken at the B1-sites. Other spectra have been offset for clarity by 0.4 units in the y-scale above the previous spectrum.

Supplementary Fig. 3a[1]. To make these definitions easier to visualize, we have also provided the site types for the nearest neighbor sites in Supplementary Fig. 3a. We have chosen to analyze the B-sites because in the sampling of sites we used to measure the local differential conductance spectra, shown in Fig. 4 of the main text, the B-sites were the most abundant. Also, in tight-binding calculations, it has been shown that B-sites exhibit different electronic behavior[2], and analyzing the B-sites in our system will allow us to directly compare the sites in our system with those in tight-binding systems. Once the B-sites were separated according to their second order vertex structures (Supplementary Fig. 3b), we normalized each spectrum by dividing by the spatially averaged spectrum of the bare copper surface, as described in the main text. In Supplementary Fig. 3c, we present the spectra averaged by each second order vertex structure. Notice that here we averaged together the spectra for the B6 site and the B7 site, since each second order vertex type

only had one site and they are the mirror reflection of each other. In Fig. 4d, we showed the spectra for the different first order vertex structures and when we calculated the variance between each of those averaged spectra and the total density of states the variance was on the order of 0.1. When we calculated the variance between the averaged spectra for each second order vertex structure shown in Supplementary Fig. 3c and the average spectra of all the B-sites, the variance was on the order of 10^{-4}. Since the variance in electronic behavior between the second order vertex structures is several orders of magnitude smaller than the variance between the first order vertex structures, we believe it is sufficient to say that the B-sites in our synthetic Penrose tiling system exhibit the same electronic behavior and we do not see forbidden B-sites which were predicted by tight-binding calculations[2].

Supplementary Note 3

Normalization of Differential Conductance

As discussed in the main text, we measured the differential conductance spectra of our synthetic Penrose tiling over a large sampling of sites. The spectra shown in the main text were all normalized by dividing each spectrum by the spatially averaged spectrum of the bare copper surface, shown in Fig. 4b. To demonstrate that the features we see in these spectra are not artifacts of the normalization method we use, we present selected spectra using another normalization method in Supplementary Fig. 4. Supplementary Fig. 4a is the same result from Fig. 4c in the main text shown between -300 mV and 300 mV to make it easier to see the individual peaks and features, and is included here for simpler comparison between the two normalization methods. The spectra in Supplementary Fig. 4b-c were normalized by subtracting a linear

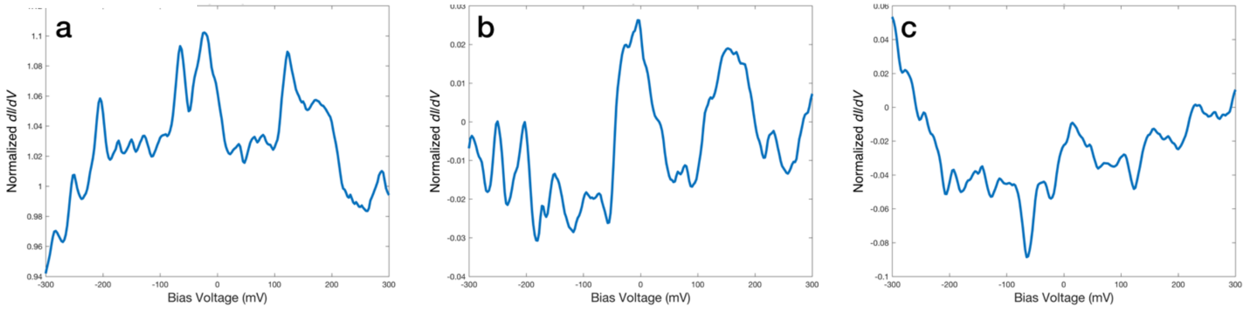

Supplementary Figure 4: Normalizing the Tunneling Spectroscopy a Different Way (a) The total normalized conductance spectra, calculated by the ratio of the average of all 61 differential conductance spectra measured in the quasicrystal and the spatially averaged differential conductance spectra of the bare Cu surface, as shown in Fig. 4c. (b) The total normalized conductance spectra, calculated by subtracting a linear fit of the spatially averaged bare Cu differential conductance spectra from the average of all 61 differential conductance spectra measured in the quasicrystal. (c) The total normalized conductance spectra of the bare Cu, calculated by subtracting a linear fit of the bare Cu differential conductance spectra from the spatially averaged bare Cu differential conductance spectra.

fit of the spatially averaged spectrum of the bare copper surface from the total normalized conductance

spectrum and the spatially averaged spectrum of the bare copper surface, respectively. We can see in Supplementary Fig. 4c that the bare copper spectrum is not exactly linear, since there are still some slight peaks in the resultant spectrum. More importantly, when we compare Supplementary Fig. 4a and 4b, we can see that while some of the smaller peaks and features have different magnitudes after each normalization and some of the larger peaks in Supplementary Fig. 4a are harder to distinguish from each other in Supplementary Fig. 4b. However, since both methods show significant peaks in the same locations, we conclude that the features we see are not due to the normalization method we have used in the main text.

Supplementary References

1. Repetowicz, P., Grimm, U. & Schreiber, M. Exact eigenstates of tight-binding Hamiltonians on the Penrose tiling. *Phys. Rev. B* **58,** 13482–13490 (1998).
2. Arai, M., Tokihiro, T., Fujiwara, T. & Kohmoto, M. Strictly localized states on a two-dimensional Penrose lattice. *Phys. Rev. B* (1988).
